# Relation between contemplative exercises and an enriched psychology students' experience in a neuroscience course

**DOI:** 10.3389/fpsyg.2014.01296

**Published:** 2014-11-18

**Authors:** Nava Levit Binnun, Ricardo Tarrasch

**Affiliations:** ^1^Sagol Center for Applied Neuroscience, School of Psychology, Interdisciplinary Center (IDC) HerzliyaHerzliya, Israel; ^2^School of Education, Special Education and Educational Counseling Department and The Sagol School for Neuroscience, Tel Aviv UniversityTel Aviv, Israel

**Keywords:** contemplative pedagogy, contemplative neuroscience, pedagogical psychology, pedagogical neuroscience

## Abstract

This article examines the relation of contemplative exercises with enhancement of students' experience during neuroscience studies. Short contemplative exercises inspired by the Buddhist tradition of self-inquiry were introduced in an undergraduate neuroscience course for psychology students. At the start of the class, all students were asked to participate in short “personal brain investigations” relevant to the topic presented. These investigations were aimed at bringing stable awareness to a specific perceptual, emotional, attentional, or cognitive process and observing it in a non-judgmental, non-personal way. In addition, students could choose to participate, for bonus credit, in a longer exercise designed to expand upon the weekly class activity. In the exercise, students continued their “personal brain investigations” for 10 min a day, 4 days a week. They wrote “lab reports” on their daily observations, obtained feedback from the teacher, and at the end of the year reviewed their reports and reflected upon their experiences during the semester. Out of 265 students, 102 students completed the bonus track and their final reflections were analyzed using qualitative methodology. In addition, 91 of the students answered a survey at the end of the course, 43 students participated in a quiz 1 year after course graduation, and the final grades of all students were collected and analyzed. Overall, students reported satisfaction from the exercises and felt they contributed to their learning experience. In the 1-year follow-up, the bonus-track students were significantly more likely than their peers to remember class material. The qualitative analysis of bonus-track students' reports revealed that the bonus-track process elicited positive feelings, helped students connect with class material and provided them with personal insights. In addition, students acquired contemplative skills, such as increased awareness and attention, non-judgmental attitudes, and better stress-management abilities. We provide examples of “personal brain investigations” and discuss limitations of introducing a contemplative approach.

## Introduction

The wealth of discoveries that neuroscientists have been making over the last three decades is driving the imagination of students from a wide range of academic fields, including the humanities and social sciences (Wiertelak and Ramirez, 2008). The appeal of neuroscience is that it offers new perspectives on human behavior and experience, making it an exciting interdisciplinary field. However, for many students in the humanities and social sciences, this new perspective is often so different from the perspectives they are used to, that the same things that had attracted them to learn neuroscience are the things that also elicit resistance (Harrington, 2013). For example, the reductionist and mechanistic neuroscience explanations often make it difficult for students to relate brain functions to their daily experiences and beliefs (Harrington, 2013). Others, especially students majoring in therapy-related fields such as psychology or social work, fail to see the relevance of significant parts of the curriculum (e.g., chapters discussing sensory and motor processes or attention) to the understanding of complex mental phenomena related to clinical work such as emotions and mood. The myriad of advances in pedagogical techniques, enhanced by technological advancements (e.g., Av-Ron et al., 2006; Brann and Sloop, 2006; Schneider et al., 2013; Schettino, 2014), have mostly suggested means to improve the teaching of theoretical neuroscience material. However, the need to arise students' curiosity and motivation and help students connect the course content to their lives and to their professional settings remains a challenge for many neuroeducators (Waldvogel, 2006; Harrington, 2013; Pollack and Korol, 2013).

Neuroeducators are not alone in this challenge. The need to complement the classical learning processes with an experiential dimension is a concern of many educators, and has led to the development of experiential learning theories (Kolb, 1984). These theories emphasize the processes of observation and reflection and the importance of *here-and-now* concrete experiences as a means to test and validate abstract concepts (Kolb, 1984). Approaches that have been developed within the experiential pedagogical framework augment immediate engagement with learned material by encouraging students to reflect upon a problem related to class material, and then enter a process of exploration and experimentation with the problem (Stewart and Stavrianeas, 2008). Indeed, extensive pedagogical experience has shown that students are more receptive to learning a topic of interest if they are allowed hands-on access to it (Allard and Barman, 1994; Colburn and Clough, 1997; Blank, 2000; Lawson, 2000; Stewart and Stavrianeas, 2008).

When coming to apply similar experiential approaches to the teaching of neuroscience, it is natural to design processes of reflection and exploration around physical objects (e.g., a slice of a rodent's brain) or using external knowledge databases (such as the Internet) to assist the exploration process of a topic. For example, Stewart and Stavrianeas (2008), developed a laboratory activity exploring issues of memory in order to enrich a neuroscience undergraduate class geared toward non-science students. In the laboratory activity, students imagined being notified that their grandmother has been diagnosed with Alzheimer's and used various databases to explore the memory deficits associated with the disease and characterize its symptoms.

Here we suggest that contemplative methods and first-person tools can expand such an experiential pedagogical approach, by enabling hands-on exploration of internal mental processes as a means to engage students in neuroscience courses. Contemplative methods cultivate inner awareness through rigorous first-person investigations. When introduced in an educational setting, these methods have been shown to encourage experiential and affective learning (Miller, 1994; Hart, 2004; Zajonc, 2006b; Brady, 2007; Shapiro et al., 2011). Indeed, recently there has been a growing interest in integrating contemplative methods into higher education (Bush, 2011; Barbezat and Bush, 2013; Zajonc, 2013) to accompany traditional ways of learning (e.g., critical or analytical reasoning). Contemplative pedagogy can range from silence at the start of the class to exercises that cultivate general mental capacities (e.g., concentration) to support the learning process. More recently, innovative contemplative practices relating to course content have been developed and introduced into courses ranging from theater to economics, philosophy and cosmology (Zajonc, 2006a). However, as far as we know, the use of contemplative tools to support neuroscience teaching has not been yet described.

In many ways, neuroscience courses are ideal for introducing contemplative methods. This is mainly due to the fact that in recent years Buddhist psychology and practices, as well as Buddhist contemplative methods of inquiry, have been gaining popularity in the fields of neuroscience and psychology (Varela et al., 1992; Lutz and Thompson, 2003; Lutz et al., 2007; Lutz, 2010; Desbordes and Negi, 2013). Mindfulness meditation, one of the central techniques used by Buddhists in their observations and experimentations on the human mind (Varela et al., 1992; Lutz et al., 2007; Dorjee, 2010; Lutz, 2010; Grabovac et al., 2011), has especially attracted the attention of the scientific community. Mindfulness can be defined as a skill set in which three attentional skills work together (Young, 2013). The first is the skill of concentration, which enables the direction and stabilization of attention toward an object of inquiry. The second is the skill of perceptual clarity toward anything that arises at the present moment. The third is the skill of holding an attitude of equanimity and “letting go” of judgments about the object of inquiry. Jon Kabat Zinn's famous sentence, “where ever you go, there you are,” (Kabat-Zinn, 1994) captures the essence of the experience of being mindful—the ability to dispassionately observe the experience of the present moment with nonjudgmental openness. According to traditional Buddhist texts the mindful awareness skill set can be directed toward the investigation of many levels of the human experience, including processes related to physical senses, emotions, thoughts, and various states of consciousness (Ekman et al., 2005; Raffone and Srinivasan, 2010; Grabovac et al., 2011).

On one hand, Buddhist methods of inquiry are similar to Western scientific methods in that they take an objective non-judgmental, non-personal stance toward their objects of inquiry. On the other hand, these methods of inquiry are directed toward the subjective self and in what underlies its subjectivity. Thus, they can bridge the gap between the neuroscientific viewpoint of the human being and the psychological one. Indeed, a new field called “contemplative neuroscience” has emerged in which neuroscientists not only study the neural correlates of various contemplative methods but attempt to combine methods of contemplation with scientific inquiry (Varela et al., 1992; Gallagher and Varela, 2003; Lutz et al., 2007; Lutz, 2010; Desbordes and Negi, 2013). Researchers in this field believe that such contemplative methods can advance scientific theories and models of consciousness, emotion and cognitive processing (Overgaard et al., 2008). Francisco Varela, one of the leading figures in this field, has claimed that contemplative tools are essential because “without embracing the relevance and importance of everyday, lived human experience, the power and sophistication of contemporary cognitive science could generate a divided scientific culture in which our scientific conceptions of life and mind on the one hand, and our everyday, lived self-understanding on the other, become irreconcilable” (Varela et al., 1992).

Importantly, the use of contemplative first-person methods as a quantitative tool in cognitive sciences is still a matter of debate (Overgaard et al., 2008). Experimental psychology has long abandoned introspective or phenomenological methods, and although contemplative methods offer a first-person approach that is based on higher levels of attentional stability, the subjective nature of first-person observations and the fact they cannot be intersubjectively verified still poses a serious hurdle (Overgaard et al., 2008). Though several attempts have been made to use first-person data to guide neuroscientific understanding (e.g., Lutz et al., 2002; Petitmengin et al., 2006; Dor-Ziderman et al., 2013), there are presently no generally agreed upon methods for first-person data collection in experimental psychology and neuroscience studies.

Despite the fact that it is still not clear how the contemplative neuroscience tools should be used in the scientific quest to understand the relation between brain and mind, we suggest here that such first-person tools, when used cautiously, can be effective pedagogical tools. Specifically, the skills developed in mindfulness awareness practice, such as enhanced awareness, attentional stability, and a non-judgmental stance toward experience, can be offered to students in a neuroscience course as a method to examine their experience in a systematic way. This kind of experiential learning can in turn arouse students' curiosity and motivation to understand the relation between mind and brain and relate theoretical class material to their daily life, enhancing the saliency of course information and leading to greater course learning and satisfaction (Waldvogel, 2006; Harrington, 2013; Pollack and Korol, 2013).

This paper contains a detailed description of how the mindfulness-awareness skill set was introduced in a semester-long advanced neuroscience class, which was taught to undergraduate students majoring in psychology in one of the universities in Israel. During the course, this skill set was developed and used to create an “experimental contemplative lab” in which students used first-person investigation tools (termed here “personal brain investigations”) to enrich their understanding of the topics learned theoretically in neuroscience courses. This “experimental contemplative lab” provided the teacher the ability to engage students in material that was presented in class (e.g., attention systems or emotion processes in the brain) in an experiential way. Namely, students contemplated upon a specific mental process (by observing its unfolding in themselves), reflected upon the experience, experimented with different aspects of the experience (for example, they compared differences in physiological responses between a happy memory and a disgusting one) and described their observations (either orally in class or via written reports outside the classroom).

In addition to triggering immediate engagement in class material, there were additional goals to the use of the “personal brain investigations”: first, to help students bridge the gap between a theoretical understanding of brain function and their own personal psychological experiences; second, to acquaint the students with the advantages and shortcomings of a contemplative method of inquiry that is not often used in contemporary pedagogy; and third, to provide students with the opportunity to experience the ability to use mental training as a means of enhancing brain-based processes they were introduced to in class, such as attention and emotion-regulation skills (Davidson et al., 2012).

Although students' reactions seemed very enthusiastic toward this pedagogical approach, we submitted it to an empirical evaluation. Based on the pedagogical framework and the goals stated above, we set out to investigate whether “personal brain investigations” would be associated with student satisfaction and enhanced course learning. Specifically, we investigated whether these exercises were associated with better final grades, with the ability of students to relate class material to their daily life and with better long-term retention of class information. We also examined whether “personal brain investigations” would relate to students' ability to appreciate the possible contributions of contemplative methods.

In order to test our research questions we undertook a mixed-measures approach using both quantitative and qualitative methods. In the quantitative analysis, the grades of all students were obtained and compared between those who participated in the bonus track and those that didn't; a short survey was given at the end of the semester to assess students' satisfaction from the exercises, the exercises' contribution to students' learning and students' attitude toward a contemplative approach; Finally, a quiz was distributed 1 year after graduation from the course to assess the effect of the “personal brain investigations” on memory of class material. The qualitative analysis was introduced in order to gain a deeper understanding of how the contemplative exercises contributed to the students. The qualitative analysis method was based on thematic analysis of students' self-reports at the end of the course.

In this paper, we describe the “experimental contemplative lab” that was created to support neuroscience teaching. We provide examples of “personal brain investigations,” along with an explication of how they related to different class topics. We then review excerpts from students' “findings” following their investigations, and report the results of our quantitative and qualitative evaluations of the process the students underwent. Finally, we discuss the benefits of using contemplative techniques in a neuroscience course and consider the conditions for this approach to be fruitful.

## Materials and methods

### Participants

The participants in this study were students that enrolled in an undergraduate psychology course entitled “Brain, Cognition, and Emotion,” an advanced obligatory neuroscience course at the Interdisciplinary Center (IDC), Herzliya, Israel, in the winter semester of either 2011 or 2012 (*N* = 124 in 2011, 89 females, age range 20–36, mean age 23.5; *N* = 143 in 2012, 98 females, age range 19–37, mean age 23.2).

### Procedure

#### Overview

Contemplative exercises were introduced in the above-mentioned 13-week course, which was taught by the first author who is a neuroscientist as well as a contemplative practitioner with a regular weekly practice and 15 years of experience in various mindfulness practices, including Buddhist Vipassana meditation, Hatha yoga practice and mindfulness-based stress reduction (MBSR, Kabat-Zinn, 2013). The exercises were in the form of “personal brain investigations” that were defined as specific investigations of mental processes, aimed at obtaining a richer understanding of these processes and their relations to other mental and brain processes. The “personal brain investigations” were conducted in two formats. The first consisted of short (10-min) weekly in-class investigations, which were done at the start of each class according to the teacher's instructions. The second consisted of longer investigations that students could choose to do for extra credit as part of a bonus track (4 days a week, 10 min a day). In these longer investigations, students summarized their daily observations, obtained bi-weekly feedback from the teacher, and wrote a final assignment, which included a summary and reflection of the entire investigation process. The evaluation of these contemplative exercises is based upon an anonymous survey that was given at the end of each semester, a short quiz that was distributed to the 2012 class a year after they completed the class, and a qualitative analysis that was performed on the summaries the bonus-track students wrote at the end of the semester.

#### Presentation of “personal brain investigations” to students

“Personal brain investigations” were defined to the students as specific investigations of their mental processes, aimed at obtaining a richer understanding of these processes and their relations to other mental and brain processes. Students were invited to take a “third-person” scientific stance and assume an attitude of equanimity and non-judgment toward the objects of investigation (mental and brain processes). It was stressed that the purpose of the investigations was not one of personal, psychological self-inquiry but rather an opportunity to have a unique view of the human experience. Students were invited to imagine that they were entering a laboratory, putting on a white lab-coat and peering into a special microscope; the fact that it was their own brain they were viewing was due to the “mere constraints of the situation.”

Students were told that “personal brain investigations” were a form of contemplative exercise. They were also given a brief account of the developing field of contemplative neuroscience and how various contemplative techniques have been gaining interest among neuroscientists, who are interested both in their neural effects and the possibility to use them as scientific methods. Importantly, the ongoing debate among cognitive scientists regarding the use of first-person methods was discussed. In addition to introducing arguments in favor of the contemplative approach and examples of attempts to use it as a scientific methodology (Jack and Roepstorff, 2002; Lutz et al., 2002; Petitmengin et al., 2006; Desbordes and Negi, 2013), the shortcomings of, and objections to, this approach were also discussed (Jack and Roepstorff, 2002; Overgaard et al., 2008). It was emphasized that although much insight can be gained from first-person investigations, their use as a scientific tool is still controversial.

#### Weekly in-class and bonus credit exercises

The “personal brain investigations” were conducted in two settings:

- *In-class investigations*: All students who attended the class were invited to participate in a “personal brain investigation” relevant to the day's subject matter. These “personal brain investigations,” which lasted 10-min and took place at the start of each class, were aimed at bringing awareness to specific functions (e.g., related to perceptual, emotional, attentional, thought, or interpersonal processes) and observing them in non-judgmental, non-personal ways. Students were not forced to participate, but were asked not to engage in any activity that might disturb other students (e.g., typing on a computer).
- *Bonus-track investigations:* Students could choose to participate, for bonus credit, in a longer exercise designed to expand upon the weekly in-class investigations. In this bonus track, students (hereafter called “bonus-track students”) were asked to continue their investigations for 10 min a day, 4 days a week, and write a weekly “lab report” on their daily observations. In each week, the exercises resembled those given in class but the prompts were often broader, containing suggestions for non-formal inquiry in daily life (e.g., “notice the things that work like magnets on your attention while you are walking on campus”). The bonus-track students' reports were reviewed by the teacher every 2 weeks via an online system. In addition to the weekly lab reports, students were given an assignment at the end of the semester to summarize their observations and insights, and reflect upon their experiences during the semester.

The only prerequisite to gain credit in the bonus track was full participation and completion of all the requirements. Students could drop out at any point without penalty. However, they could not stop the longer exercises and then return to completing them. Students were explicitly told that there were no correct or incorrect answers, as anything that might arise (including difficulties and resistance to the exercise itself) would be legitimate, so long as the investigation process was occurring and awareness was brought to it. Of the students enrolled in the courses, 56% (*N* = 70) of students from the 2011-class joined the bonus track and 59% (*N* = 84) did so in 2012. Seventy percent of the joining students (*N* = 49, 41 females) completed the bonus track in 2011 and 63% (*N* = 53, 46 females) did so in 2012.

#### Ongoing review of bonus-track students' lab reports

Once every 2 weeks the teacher provided students in the bonus track with feedback on their work. The teacher endeavored to take a non-judgmental stance toward the students and encouraged each to maintain a non-subjective, non-judgmental attitude. On occasion, the teacher suggested further inquiry regarding interesting observations or told them to keep up with the good work. Bonus-track students were requested to write short, technical reports beginning with sentences like “I noticed that X happens when Y happens.” They were also asked to write in a non-personal tone (e.g., “I noticed thoughts can stimulate feeling X” rather than “I have thoughts that make me feel X”).

#### Examples of “personal brain investigations”

Both in class and at home (for the bonus track), the first few investigations and the beginning of all subsequent investigations were dedicated to the development of concentration as well as equanimity skills (i.e., developing personal tools for investigation). Students were asked to bring their attention to their breathing process and to count the number of breaths they were able to be fully aware of before becoming distracted. When a person had realized his/her attention had wandered and was no longer focused on the breath, s/he should re-start the counting and try to maintain awareness for a greater number of breaths. They were encouraged to see this as a “polishing the microscope lens” exercise and be non-judgmental toward their success or failure. Subsequent investigations always began with a few minutes of the “polishing the microscope lens” exercise, wherein students attempted to reach the count of 10 full breaths without becoming distracted. Interestingly, even the “polishing the microscope lens” exercise provided many opportunities for investigation and discovery (for example, examining what made it so difficult to be completely aware of 10 full breaths).

Several examples of “personal brain investigation” are listed below (see Supplementary Section for additional examples).

***Example 1: Investigating magnets of attention***. This investigation was conducted at the beginning of a class about the ventral and dorsal attention networks in the brain, which are dedicated to the detection of novel stimuli and the focusing of attention, respectively (Corbetta and Shulman, 2002; Purves, 2008). Students were invited to conduct the “polishing the microscope lens” exercise for 10 min while noticing the internal experiences, as well as the external objects, that diverted their attention away from a focus on the breath. Students' findings were shared in class and later discussed in the context of the ventral and dorsal attention networks.

In the same week, students in the bonus track expanded this investigation at home in four 10-min daily exercises. They were encouraged to investigate which types of stimuli (e.g., human voices vs. mechanical noise) captured their attention more easily, and how different emotional and arousal states influenced their ability to focus their attention on the breath. They were encouraged to continue this investigation in different life-settings (e.g., walking in the park, sitting in the cafeteria).

***Example 2: Investigating the relationships among emotions, sensations, and thoughts***. This investigation was conducted at the beginning of a class discussing theories of emotions (e.g., the James-Lang, the Cannon-Bard, the Schachter-Singer and the Cacioppo theories; Freberg, 2009) and the attempts of these theories to address the relationships among emotions, physiological processes, and cognitive evaluations. Following a short (3-min) “polishing the microscope lens” exercise, students were asked to direct their attention toward their emotional feelings and notice the bodily sensations that accompanied them. They were then asked to remember a pleasant memory and to notice both the emotional feeling and the bodily sensations that accompanied it. The same instruction was repeated for an unpleasant memory. They were instructed to be as precise as possible, noticing where exactly the sensations arose, as well as their temporal sequence, intensity and valence. This whole procedure lasted about 10 min following which students shared their observations in class.

In the same week, the bonus-track students continued this investigation at home while receiving additional instructions. The group was asked to notice the order in which emotions, thoughts, or bodily sensations arose—and how the order changed on a daily basis based on mood or external events. As a “non-formal,” optional inquiry they were asked to choose one transition a day (e.g., entering/exiting the college, entering/exiting their house, entering/exiting their car) and to stop for a few seconds to notice the state of their bodily feelings and how it was affecting their mood and behavior.

***Example 3: Investigating emotional valence***. This investigation was conducted in the beginning of a class that dealt with the valence hypothesis in relation to brain lateralization (Purves, 2008; Miller et al., 2012). Following a short “polishing the lens” process, students were asked to direct their attention toward their emotional feelings and notice whether the bodily expression was “pleasant,” “unpleasant,” or “neutral.” After 10 min, students shared their observations with the class.

In the bonus track that week, students were asked to deepen their investigation, and notice changes in the valence of sensations and emotions during each of the four daily inquiries, as well as whether sensations and emotions fluctuated on a daily basis based on mood or external events.

***Example 4: Investigating the relation between intrinsic and extrinsic processes***. This investigation was conducted in a class that dealt with the brain's intrinsic network (also known as the “default state” network; Raichle and Snyder, 2007; Purves, 2008, p. 321). Students were taught that the default network tends to deactivate when an extrinsic network (also known as the “task positive” network) is activated (Raichle and Snyder, 2007), and that it has been related to autobiographical memory and to stimulus-independent thought processes and mind-wandering (Mason et al., 2007). In class, therefore, students were asked to notice and compare the appearance of internally evoked thoughts and emotions in two different situations—when focusing on their breath (a relatively passive task) and when counting backwards from 100 in steps of 7 (a relatively difficult task necessitating additional attention resources).

That week, the bonus-track group was asked to deepen this investigation and observe the rate of occurrence of internal processes (e.g., thoughts and emotions) in a variety of tasks that differed in the type and amount of effort required. They were asked to notice if, and in what way, the appearance of internal processes affected the activity they were engaged in. Tasks included focusing on the breath, counting backward from 100 in steps of 7 (as we did in class), focusing attention on one's feet (and trying to notice the subtle sensations that arose), and focusing on the subtle details of a movement task (e.g., bringing the hands back and forth above the head to touch each other). As a non-formal, optional inquiry students in the bonus track were asked to pay attention to the tasks that completely absorbed them, and to those that allowed some internal processing.

### Evaluations of the “personal brain investigations”

#### Ethical approval

Following approval by the local ethics committee, informed consent was obtained from students for using their bonus-track summaries, survey answers and quiz results for the evaluation of the pedagogical method they had experienced in class.

#### Descriptive statistics of students

When analyzing the grades obtained by students in the basic neuroscience course that was taught in the previous year (entitled “The physiological basis of behavior”) by the same teacher, we found that students that participated in the bonus track program obtained a significantly higher grade in the course (*M* = 88.01, *SD* = 5.79) as compared to students that did not participate in the program (*M* = 85.85, *SD* = 7.57). In other words, students that participated in the bonus program had *a priori* significantly higher grades as compared to students that did not enroll in the program [*t*_(225)_ = 2.32, *p* < 0.05, Cohen's *d* = 0.32]. In spite of this fact, the bonus-track group was not composed only of strong students. When splitting the students based on their median grade from the previous year into two groups, 36.8% of students who enrolled in the bonus track program were below the median, while 46.1% of students were above the median [the difference in percentages being non-significant (χ^2^_(1)_ = 1.99, *p* = 0.16)]. Table 1 describes the number of students participating in the various measures described here and below.

**Table 1:** **Number of students used in various measures**.

	**Overall**	**Joined bonus track**	**Completed bonus track**	**Filled survey**	**Filled quiz**	**Grade**	**Qualitative evaluations**
2011	124	70	49	42		103	49
2012	143	84	63	49	43	124	53

*The table describes for each year, the number of overall number of students that participated in the class, the number of students that joined the bonus track, the number of students that completed the bonus track, the number of students that filled the survey, the number of students that participated in the quiz, the number of students for which grades were obtained, the number of students that their final reports were submitted to qualitative analysis*.

#### Quantitative evaluations

***Survey***. At the end of the semester a research assistant distributed an anonymous survey to all students who were present in class (*N* = 42 completed the survey in 2011 and *N* = 49 in 2012; classes were not obligatory, but by the end of the semester approximately ~70 students were attending each class). The survey related to both in-class and bonus track brain investigations. Students were asked to report the number of brain investigations they participated in during class and whether they participated in the bonus track. Then, for each category (weekly investigations and bonus track), students were asked to rate on a 5-point Likert scale their satisfaction with the personal brain investigations, how much they felt they had learned from them, and whether they would use such a contemplative approach in the future. Since the bonus-track group invested much effort in the personal brain investigations we also asked how important they believe it was to combine these investigations in the training process of psychology students. For assessing the overall impact of each rating, the averages of each scale were compared to the midpoint of the scale (*M* = 3) using one-sample *t*-tests.

***Quiz at 1-year follow-up***. In addition to assessing student satisfaction, we were interested in examining whether our contemplative approach contributed to the long-term retention of class material. With this aim, we distributed a quiz at the beginning of another undergraduate psychology course, “Abnormal Psychology,” which is obligatory and taken by all students the year after the “Brain, Cognition, and Emotion” course. The aim of this quiz was to assess how much they remembered from the previous year's “Brain, Cognition, and Emotion” class. This follow-up quiz was given only to students from the 2012 year, and only to those who were present in class on that day. The quiz contained 10 multiple-choice questions taken from previous exams as well as a short questionnaire assessing whether students participated in the brain investigations in class and in the bonus track. We also requested their self-assessment of the amount of classes they attended (0–25, 26–50, 51–75, 76–100%), their level of participation in class in general (below average, average, above average), and their level of concentration in class (low, medium, high). In addition, we asked them to indicate the range of their final course grade (0–60, 61–70, 71–80, 81–90, 91–100). Overall, 43 students completed the quiz; all responses were anonymous. We first compared students who indicated that they participated in the bonus track (*N* = 22) to those who indicated that they did not participate in the program (*N* = 19). Two students who indicated that they participated in a partial manner in the bonus track were not included in the bonus track analyses. As the variances of the groups did not significantly differ [*F*_(1, 39)_ = 0.225, *p* = 0.638], the groups were compared using a *t*-test for independent samples assuming homogeneity of variances. We then compared students who reported participating at least partially in the in-class investigations but not in the bonus track (*N* = 9) to those who reported that they did not participate at all in any investigation (*N* = 10). In this case, too, the variances of the groups did not significantly differ [*F*_(1, 17)_ = 0.002, *p* = 0.970], and the groups were compared using a *t*-test for independent samples assuming homogeneity of variances.

#### Qualitative evaluations

In the last week of the semester, the bonus-track students were given a “final project” in which they were asked to review all of their findings throughout the semester and summarize the 10 most interesting insights from the process. In addition, they were asked to reflect upon the process in general and to describe their experiences. General guidelines were provided for this feedback: “Please reflect upon your experience, e.g., was it interesting, enjoyable, boring, did you do it because it was obligatory, what would you change, what was most significant, what was least interesting. Please provide additional comments and suggestions that would help us improve in the future.” Importantly, students knew that if they completed all the lab reports they would automatically receive full bonus credit, as there were no correct answers.

The reflections and summaries from the 2011 and 2012 bonus-track students were merged together (total *N* = 102). They were analyzed through a thematic analysis grounded in a social constructionist framework (Denzin and Lincoln, 1994), which Braun and Clarke (2006) described as a beneficial method for identifying, analyzing, and reporting patterns (themes) within data. To analyze the summaries, three coders (the first author, who was also the teacher; the second author, who was not related to the development or dissemination of the program; and a psychology student with previous experience in qualitative coding, that was naïve to the aims of the manuscript and was paid for her efforts) first independently read 10 out of the 102 summaries at least twice each, to become familiar with the content and to isolate sentences in an attempt to name and classify central themes. Each sentence could be coded for more than one theme or subcomponent. The three coders then shared their impressions of the themes that arose. In order to reduce bias, a significant effort was made to avoid trying to convince the other coders that a given theme was correct; instead, there was an emphasis on grouping together, in broader categories, themes that seemed to relate to the same phenomena.

We sought to identify general categories or recurring patterns that could depict a well-fitting, data-driven “story” of participants' experiences. General categories included contribution to the learning process, contribution to personal development, technical aspects of the process, and so forth. We then compared these different general categories to identify possible similarities, thereby enabling the construction of six core themes that each blended several general categories. After the categories were formed, two of the coders analyzed 20 more summaries; additional sub-categories were added and the three coders discussed disparities. Ten more summaries were rated again by two of the coders reaching an average item concordance Kappa between the raters of 0.91. Subsequently, the rater that was not one of the authors coded all remaining summaries, and these classifications served as the data for subsequent analysis. We made extensive use of *in vivo* codes (Strauss, 1987), drawn from the participants' own accounts in ways that attempted to summarize participants' own meanings in their own words.

## Results

### Examples of students' observations and findings

Examples of the weekly reports and end-of-semester insights by bonus-track students appear in Table 2.

**Table 2:** **Examples of student observations in the personal brain investigations**.

**Concentration training—“Polishing the microscope lens”**
• Sometimes I can think that I am focusing attention and concentrating and then discover that I wasn't and that there was a whole part of the experience I missed.
• I noticed that in the mornings it is easier for me to focus attention and concentrate. There are less thoughts and their rate is slower.
• I noticed that the attention system prefers steady objects to focus upon and less dynamic, such as a steady noise, or looking at a still object.
• I noticed that when attention is focused on the breath and the body the physical system becomes calmer.
• Expectation and motivation to succeed can distract attention.
• Repeating the same exercise influences attention levels. In other words, when training several times on the same exercise, the levels of attentions and concentration improves from session to session.
• Attention to something can silence another feeling/thought, for example, attention to thoughts silences the external world and the “noise” that accompanies it.
**Investigating magnets of attention**
• I noticed that my emotional state influences my ability to concentrate. When my arousal is high (for example in a state of anger or excitement) it is harder to focus.
• I noticed that when my attention is focused on a sensory stimulus or is temporarily distracted by a sensory stimulus, it is relatively easy to bring the attention back and refocus. When the emotional system is activated by an emotion or thought it distracts the ability to focus. After that it is very hard to refocus attention.
• When attention is distracted to an external stimuli, sometimes a physiological and an emotional response accompanies it and sometimes it doesn't.
• When the external stimuli is passing and transient (cars passing or honking), attention quickly returns to the breath and internal concentration. But when the external stimuli is continuous (car engines, people talking) it is hard to bring attention back to the breath.
**Investigating the relationships between emotions, sensations, and thoughts**
• I noticed that thoughts stimulate feelings that involve bodily sensations. [Also] I noticed that bodily sensations (for example, unpleasant sensation or pain in a certain area) can stimulate feeling and thoughts.
• When the trigger is an internal physiological sensation in the body (for example, a sensation of pain, even very light pain), there arises an unpleasant feeling of pressure in the stomach. After that comes thoughts that are quite “technical” and are related to that bodily sensation (Why am I in pain?).
• When the thoughts were “neutral” general thoughts about life, thoughts that come and go, I didn't notice any physical sensation that accompanied them. When the thoughts were disturbing thoughts (something I did wrong, something I need to do), there was an unpleasant sensation in the chest that accompanied them.
• I noticed that in a transition from one environment to another the body tries to adapt to the state of the new environment, e.g., the weather. For example, [the body contracts] before entering a state that is cold.
• I noticed a connection between the sensory system and the emotional system, so that when I feel a sensation, immediately certain feelings arise which then enhance the sensory sensation, and so on. A bi-directional route.
• I noticed that the mood I start the day with has a significant influence on my attention system, my thoughts throughout the day, and my feelings and physical sensations.
**Investigating emotional valance**
• I noticed that the attention system is influenced more by negative stimuli than by positive ones and it's more difficult for it to detach from the negative stimuli.
• I noticed that when I move my awareness [and concentration] to a certain body area, [a feeling of restfulness] develops there. Attending to certain areas leads to a pleasant sensation in an itchy area.
• I noticed that focusing on a painful area, being in that moment with the pain, not fighting it and just accepting it leads to a reduced sense of pain.
• I noticed that focusing on a certain area enhances and sharpens the bodily sensations in it.
• Negative emotions narrow down the attention beam and positive emotions widen it.
**Investigating the relation between intrinsic and extrinsic processes**
• When attention is [needed for] a task that requires a skill or knowledge like arithmetical calculation, it is hard to keep focused [on the breath], and high chances that attention will be [entirely] diverted to the skill required in the task.
• The type and difficulty of the task influences a person's attention level and focus. When the task is challenging, we concentrate on it, and it would not be easy to shift our attention to less challenging tasks.
• I notices that when counting back most of my attention resources are allocated at the counting itself. The only times I suddenly noticed the feeling of my body's posture was when the subtraction of the two numbers was easy for me.
• I noticed that every thought or feeling slowed down my ability to count-back.
• In a world full of stimuli it is easy to get “absorbed” in them and forget about yourself.

*These examples were taken from students' weekly “lab-reports” as well as from their list of insights that they submitted in the end of the semester (after they went over all their lab reports and reflected upon the whole process). Here we only give examples for the investigations we mentioned in the Methods Section. For more examples of investigations and observations—see Supplementary Section*.

### Quantitative assessment

#### Grades

Students that participated in the bonus track program (*M* = 84.46, *SD* = 9.23) obtained a significantly higher grade in the course [*t*_(228)_ = 2.01, *p* < 0.05, Cohen's *d* = 0.28] as compared to students that did not participate in the program (*M* = 81.17, *SD* = 13.91). However, when taking the grades they obtained in the previous year (see “Descriptive statistics of students” section above) as covariate the difference between the groups, in terms of the final grade in the advanced neuroscience course, became non-significant [*F*_(1, 224)_ = 0.45, *p* = 0.501, partial Eta squared = 0.002].

#### Survey

Of the students who answered the survey (*N* = 90), 71% (*N* = 64) participated in the bonus-track. Of the remaining students (who did not participate in the bonus-track), only 11 students reported having participated in most or all brain investigations.

Table 3 displays the average bonus-track student ratings for the in-class weekly investigation—for how much satisfaction they gained from the weekly investigations and how much they learned from them, and how much they would use contemplative tools in the future. One-sample *t*-tests assessing the average ratings of the three scales yielded significant results (all *p*'s < 0.001) when compared to the midpoint of the scales (*M* = 3), indicating significant levels of satisfaction, learning, and intention to use contemplation tools in the future. Despite the low n, we performed a similar analysis for the 11 students who did not participate in the bonus-track but reported participating in most or all investigations. Although average scores for satisfaction (*M* = 3.45) and future use of contemplative tools (*M* = 3.29) was greater than the midpoint, they were not significantly different (*p* > 0.27).

**Table 3:** **Ratings of satisfaction, learning, and future-intention to use contemplation tools following in-class weekly investigations, among bonus-track students**.

	**Mean (scale from 1 = lowest to 5 = highest)**	**Standard deviation**	***N***
Grade the rate of satisfaction you obtained from the investigations.	3.84	0.99	64
How much do you feel you learned from these investigations?	3.75	1.02	65
Do you think you will use the contemplative tools you received in this course in the future?	3.75	1.00	65

*High average ratings were obtained, compared to the midpoint of the scales (One-sample t-tests, all p's < 0.001)*.

Table 4 displays the average ratings of the bonus-track students for the bonus-track investigations—for how much satisfaction they gained from the bonus track investigations, how much they learned from them, how much they would use contemplative tools in the future and how important it was to use “personal brain investigations” in the training process of psychology students. The average ratings were again compared to the midpoint of the scale (*M* = 3) using one-sample *t*-tests. The four analyses yielded significant results (all *p*'s < 0.001).

**Table 4:** **Ratings of satisfaction, learning, future-intention to use contemplation tools and importance to combine brain investigations in the training process, among students who participated in the bonus track investigations**.

	**Mean (scale from 1 = lowest to 5 = highest)**	**Standard deviation**	***N***
Grade the rate of satisfaction you obtained from the investigations.	4.00	0.90	65
How much to you feel you learned from these investigations?	4.08	0.87	65
Do you think you will use the contemplative tools you received in this course in the future?	3.89	1.04	64
How important is it, in your opinion, to combine “personal brain investigation” in the training process of psychology students?	4.13	0.93	68

*High average ratings were obtained, compared to the midpoint of the scales (One-sample t-tests, all p's < 0.001)*.

#### Quiz

Out of the students who participated in the quiz, 91% of those who reported they had participated in the bonus track also reported to have participated, at least partially, in the in-class investigations. Bonus-track students were not different from students who did not participate in the bonus-track in their reported final grade range [*t*_(39)_ = 0.49, *p* = 0.63], reported class attendance [*t*_(39)_ = 0.764, *p* = 0.45], reported level of participation in class [*t*_(39)_ = 0.985, *p* = 0.32] and the reported level of concentration [*t*_(39)_ = 0.71, *p* = 0.48]. When comparing the results on the quiz, bonus-track students (*M* = 5.82, *SD* = 1.37) answered significantly more correct answers as compared to students who did not participate in the program (*M* = 4.68, *SD* = 1.25) [*t*_(39)_ = 2.76, *p* = 0.009, Cohen's *d* = 0.87, partial Eta squared = 0.163]. These results in the quiz remained significant even after controlling for the difference between the groups in their final advanced course grades [*F*_(1, 37)_ = 8.35, *p* = 0.006, partial Eta squared = 0.184], and also after controlling for their final advanced course grades, the percentage of classes attended, participation during class and concentration [*F*_(1, 34)_ = 6.13, *p* = 0.018, partial Eta squared = 0.153], as demonstrated by analyses of covariance (ANCOVA). Despite the small n's we also compared quiz results for students who did not participate in the bonus-track but did participate, at least partially, in the in-class investigations (*M* = 4.89, *SD* = 1.27), with those that did neither (*M* = 4.50, *SD* = 1.27). No difference in correct answers was found between the groups [*t*_(17)_ = 0.67, *p* = 0.51].

### Qualitative assessment

**Figure 1:**
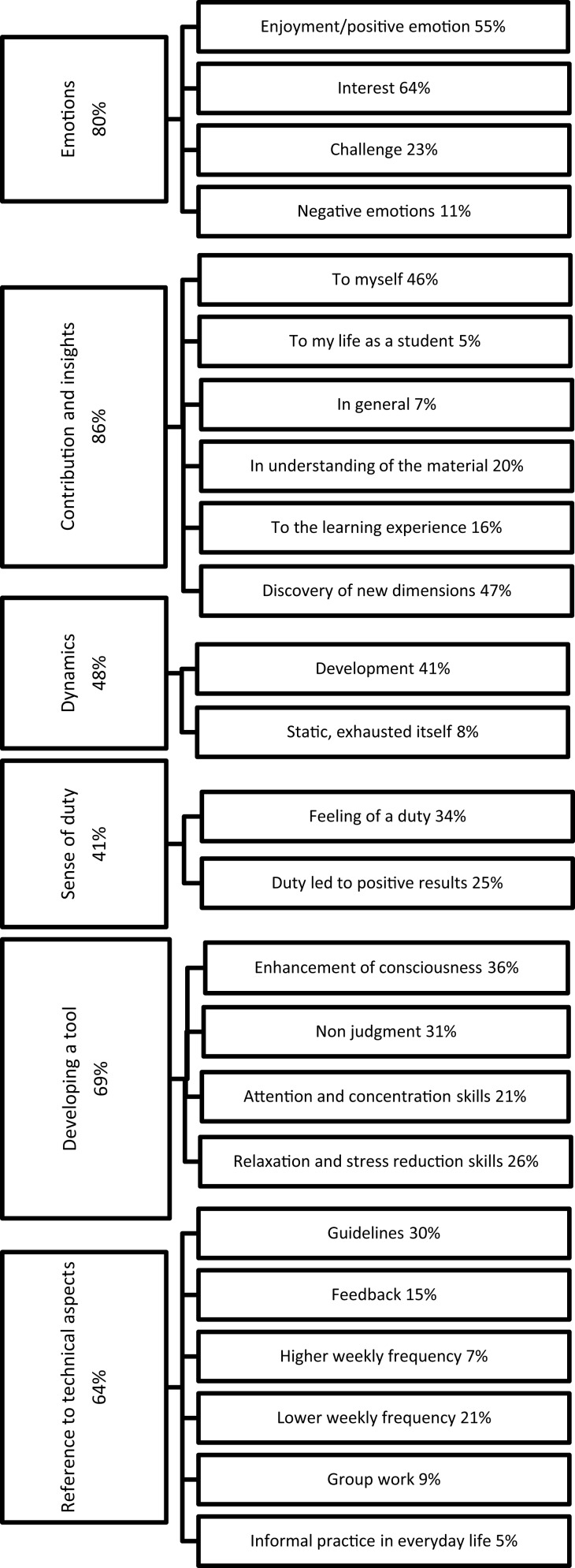
**The six core themes identified in the qualitative analysis**. Presented are also the sub-categories for each theme, with the corresponding percentage of students who included them in their reports.

When performing a thematic analysis on the summaries and reflections that the bonus-track students wrote at the end of the semester, we revealed several core themes. These included: emotions, contribution and insights, dynamics, sense of duty, developing a tool, and reference to technical aspects. Each of these core themes and their sub-categories are presented below (see Figure 1). Due to lack of space only a few quotes from students' summaries are provided for each sub-category. A full list of examples is given in Table S2 in the Supplementary Section.

#### Emotions

The first core theme that appeared in most of the students' comments (80%), related to emotions elicited by the process.

***Enjoyment/positive emotion***. A majority of students (55%) mentioned either feeling enjoyment or some other positive/enriching feeling in relation to the investigation process: “the bonus-track was a daily pause for me, to stop and look at things that interest me but I don't always have the time to investigate and learn about myself. The first time I did it was for the experience, the second investigation I did for the grade, but all the subsequent investigations I did because of enjoyment and curiosity” (Student 97). Student 84 wrote: “I enjoyed performing most of the tasks and noticing the changes and the things that are happening.”

***Interest***. A majority of students (64%) reported feeling the process they had undergone during the semester was interesting: “It was very interesting to discover new things about myself and about phenomenon that happen while directing attention in various ways and during daily activities that I preform (Student 39). Student 79 wrote: “Along the way I came across some interesting and fascinating exercises that stimulated the desire to explore, understand and learn.” Student 86 wrote: “The process was very interesting. I tried to explore and discover new things and there were times when I was indeed very surprised about what I found.”

***Challenge***. Some students (23%) reported that the process included a challenge and was effortful: “Attention is a very elusive creature that depends upon situations in a very dynamic manner” (Student 12). “I found it really hard for me to release control and focus my attention. Maybe this frustration is what led to my extra effort in the practice of the exercises” (Student 42). Student 98 wrote: “At first the tasks were very difficult; I found it hard to focus my attention.” Student 100 wrote: “Exercises that involved attending to specific body organs were more difficult for me.”

***Negative emotions***. A small number of students (11%) reported negative emotions: “I felt discomfort” (Student 56). Student 90 wrote: “The total detachment from distractors and the silence created unpleasant and negative feelings.” Student 4 wrote: “The experience was interesting in part, but I did not connect to many practices and they bored me (the practice was technical).”

#### Contribution and insights

The second core theme related to contributions and insights that the investigation process elicited and appeared in most of the reports (86%).

***Contribution to myself***. About half of the students reported a personal contribution (46%): “I discovered a lot of new things about myself. I revealed a new perspective, instead of looking outwards, to focus inwards. That is something that I never experienced personally before. This is a very instructive and enriching experience. Most of the learning was about myself, about the rich world and the interaction between the two” (Student 10). Student 11 wrote: “Although the aim of these investigations was not to look at myself and or to improve myself, I think they mostly contributed in this dimensions. I think that however I thought I was aware of my feelings beforehand, I am bit more aware of them today.”

***Contribution to my life as a student***. A few students (15%) noted contributions to them as students in general (not specifically related to the neuroscience class): “the exercises enabled a “time out” for self-observation that was very needed in this over-loaded semester” (Student 31). Student 24 wrote: “I felt that the ability to breakout from the pressure of life in general, and school in particular, and to focus on these investigations, was a present and induced a more relaxed and calm atmosphere.”

***Contributions in general***. Some students (7%) reported a broadly general contribution: “It contributed to understanding the various physiological and psychological effects through actual experience” (Student 82). Student 62 wrote: “[There was] a legitimacy to look inside and sense things that I wouldn't bring to mind if it wasn't for the brain investigations. Just as we study the psyche, it is important to learn on our bodies and ourselves the connection between body and mind.”

***Contribution to understanding of the material***. About fifth of the students (20%) reported a better understanding of the material: “I think that the brain investigations give a different perspective to the curriculum which is a unique pedagogic approach” (Student 82). Student 14 wrote: “In general I believe that a good learning process needs to include some personal experience that relates to the learned material, and I think that this process enables this. Such an experience provides depth to the contents of the course and enables a completely different learning experience from the common one. We are used to receiving information from the teachers and books; such a personal experience enables a completely different level of processing.”

***Contribution to the learning experience***. Some students (16%) reported a contribution to their learning experience: “I think the idea to enable an experiential dimension along the course is positive and very much needed especially in a psychology degree where everything is so theoretical. This enables reflecting on the class material beyond reading class summaries and enables feeling connected with the class material. They should consider doing this in other courses too” (Student 28). Student 22 wrote: “This training contributed to me personally and made me think about a lot of things that I never thought about in the past. It really added to the learning experience in this course and helped in the understanding of the material.” Student 41 noted: “It contributed to learning. To experience something is not just like reading or learning about it.”

***Discovery of new dimensions and insights***. About half the students (47%) reported the discovery of new dimensions in relation to brain and mental processes and their relation to their environment: “it was the first time I noted the actions that I do, the physical activity that is usually automatic, I felt every movement and investigated how they are done. I think I had experiences that I wouldn't have had an opportunity to investigate in any other place” (Student 17). Student 22 wrote: “I think this exercise contributed also personally and caused me to think about things I never thought about in the past.”

#### Dynamics

The third core theme related to the nature of the students' experiences—whether they reported a dynamical experience, which evolved and changed throughout the semester or a static one. Mentioning of this appeared in 48% of the summaries.

***Development***. A little less than half of the students (41%) mentioned the presence of a dynamic process that developed and evolved throughout the semester: “I think that the exercises had a developmental trajectory, therefore there is not a single exercise that I would give up” (Student 8). Student 67 noted: “While summarizing the tasks that I submitted, I was amazed to realize the process that I went through week after week.” Student 58 wrote: “At first I didn't realize how such simple exercises can bring great insights. However, with the passage of time and while performing more and more exercises, I was surprised to find how much you can learn from something so simple.”

***Static, exhausted itself***. A number of students (8%) reported feeling that the process did not develop and was static most of the time. Student 3 wrote: “Most exercises were really interesting, but there were times that I felt a sense of exhaustion and did not understand why we have to practice the same exercise again.” Student 9 wrote: “The content of the trainings was very similar most of the times, so the exercises became sometimes tedious and ineffective.”

#### Sense of duty

The fourth core theme related to a sense of duty and obligation evoked by the process and appeared in 41% of the summaries. We divided them to those that explicitly mentioned feeling of duty to those that mentioned the duty in relation to positive outcomes.

***Feeling of a duty***. About one-third of the students reported a feeling of duty (34%). However, only 17% reported a sense of duty without also reporting positive results: Student 72 wrote: “There were times when I was stressed and performed the exercises out of duty, but not in most cases.” Student 53 wrote: “The experience was mostly interesting, but at its end I felt I was doing it from a feeling of duty.” Student 93 wrote: “There were several times when I felt a sense of duty and therefore, in those times I connected only during exercise itself.”

***Duty led to positive results***. A quarter of the students (25%) reported feeling that the duty had positive outcomes. Student 68 wrote: “Without the [repeated] practice I would have never performed exercises of this type, because normally we relate to the body as a machine without needs.” Student 46 commented: “I understand that it is important to practice a large number of times in order to obtain results and insights.” Student 83 wrote: “I would not give up to the fact that weekly assignment had to be submitted on the same day and at the same time every week. The fact that the investigations were performed continuously for more than 2 months, made the process deep and interesting.”

#### Developing a tool

The fifth core theme related to the development and acquisition of some tool or skill and appeared in 69% of students' reports.

***Increased awareness***. A third of the students (36%) noted an increased awareness: “It is nice to raise the level of awareness to things that without the exercises I wouldn't necessarily be aware of” (Student 5). Student 21 wrote: “I realize I have developed a skill… now, occasionally, there are moments that I suddenly notice details I have not noticed in the past. This happens every few days, but it is interesting and also gives a good feeling.” Student 10 elaborated: “The exercises provided me with a different perspective, helped me become aware of myself, of my body, of my breath, of my movement, of how various stimuli distract me from experiencing my inner experiences.”

***Non-judgment***. A third of the students (31%) reported feeling that they had developed the ability of looking at things in a non-judgmental way, enabling a different perspective at things. Student 45 wrote: “I let myself investigate all the small things that constitute me, without trying to fix it. To try as much as I can to investigate, to focus and polish the lens.” Student 94 wrote: “I became less and less judgmental toward myself, even in advanced exercises which I did not succeed. This is an achievement by itself; to be in the process and not necessarily reach the target. The result is not less interesting.”

***Attention and concentration skills***. A fifth of the students (21%) noted the development of better attention and concentration abilities: “my ability to focus attention improved or at least rose to a higher level of awareness” (Student 18). Student 19 mentioned: “with time, attention allocation become more automatic, without relation to the specific instructions for the week.” Student 39 wrote: “the order of the exercises was meaningful, since as the weeks past I felt I became more skillful in focusing attention, and I could do more complex tasks that I assume I couldn't have done in the first weeks.”

***Relaxation and stress reduction skills***. Another ability that some students (26%) reported as the result of the bonus-track process related to ability to reduce, regulate, and cope with stress. Student 82 wrote: “I am an anxious person by nature. I and my close relatives can attest that lately the exercises helped me significantly in relaxing.” Student 47 commented: “Unfortunately, due to the large load and amount of tasks that we get, we do not always have the time to stop and breathe and be with ourselves. The exercise was a good attempt to allow us such quality time.” Student 29 wrote: “I felt this helped me to deal with stress and various feelings. In addition, it was interesting to connect to myself and just be in peace and serenity with myself for 10 min every day.”

#### Reference to technical aspects

The sixth and last core theme, which appeared in 64% of the summaries, related to technical aspects of the process and suggestions for improvement.

***Guidelines***. A third of the students (30%) commented on the guidelines. In general, students reported feeling the guidelines were important: “The practice at the beginning of class, was very powerful and gave us an introduction to what is expected from us, how to work during the week” (Student 33). “I would leave [for future bonus-track exercises] the slight vagueness in which the guidelines were given… and the explanations about polishing and focusing the lens in the first weeks that helped perform the exercise subsequently” (Student 61). Some students felt they needed even more precise instructions, such as Student 77: “I think I'd sharpen more the instructions, especially at the first few weeks and I'd love to get a bit more detailed feedback.”

***Feedback***. Some students (15%) specifically mentioned the feedback provided by the teacher: “I liked the fact that you gave feedback, even just the word “thank you”; it helped me to know that I am doing things right and that you really care and are interested in my investigations” (Student 69). Student 74 wrote: “I would continue [for future students] the persistence to investigate the processes with an “investigator's eye” and not from a psychological perspective.” Student 95 commented: “I would add feedback in the form of questions. Once you asked me what I meant when I said “soul.” This question made me think beyond, and contributed to my process.”

***Higher weekly frequency***. A few students (7%) reported they would have liked a higher frequency of exercises (i.e., more than 4 times a week): “I wouldn't change the fact you need to report 4 observations a week because every observation enables you to look at the exercise from a different perspective and in that way discover more” (Student 59). “I would stay with four weekly observations since there are real difference when you perform the exercise in different places, different moods, different hours, etc.” (Student 78).

***Lower weekly frequency***. A fifth of students (21%) reported they would have preferred a lower frequency of exercise: “I think that it was enough to report only three times a week since the fourth time was already too much of a burden and sometimes made me forget the main reason for doing the exercise” (Student 18). “Although the exercises are only 10 min a day, I felt the requirement to do them four times a week was a bit exaggerated” (Student 48).

***Group work***. A few students (9%) commented on the benefits of group work: “I would set at least two exploration tasks to work on pairs so we can see how the same questions and guidelines are interpreted and experienced differently by each person. It is important that we also see the inter-individual differences” (Student 97). “I would have been happy if once all the participants in the bonus-track can meet and share experiences, I think this could have been interesting” (Student 14).

***Informal practice in everyday life***. Only 6% of the students mentioned the informal practices that were given as additional suggestions for everyday life. Student 21 wrote: “I think the informal practice helped me much as it enabled me to see how the investigations are reflected in daily life.” Student 50 wrote: “It is important [for future students] to continue with the informal practices that for me, at least, enabled the most interesting discoveries.”

## Discussion

Contemplative pedagogy is becoming increasingly popular and has recently been introduced to a variety of subjects ranging from poetry to medicine to law (Zajonc, 2013). Inspired by Buddhist contemplative methods of investigation and the emerging field of contemplative neuroscience, we suggested a way of introducing methods of contemplative pedagogy into the teaching of neuroscience in the form of an “experimental contemplative lab” with “personal brain investigations.” We provided examples of short 10-min investigations that students were asked to do in the beginning of our weekly neuroscience class, as well as longer weekly investigations they could perform at home. These investigations enabled students to focus awareness and attention in a non-judgmental way to sensory, emotional, motor, cognitive, motivational, and arousal processes, and even to awareness and attention themselves—all brain processes that are taught in advanced neuroscience courses.

Using both quantitative and qualitative measures, we evaluated the contribution of these contemplative investigations to course learning and to student satisfaction, as well as to the ability to appreciate the usefulness of contemplative tools.

Our quantitative evaluations revealed that students who performed both the short in-class investigations and the longer bonus-track ones, expressed significant satisfaction from both formats of investigations and reported feeling the investigations contributed to their learning process. All these students also reported that the contemplative tools they acquired might be useful to them in the future. Bonus-track students also reported feeling that it is important to combine similar exercises in the training of psychology students in general. A similar trend was also obtained for students who participated in the in-class investigations only, albeit not significant, probably because of the low statistical power due to the small number of students available for this analysis. Analyzing final grades of all students revealed that students that chose to take the bonus track were *a priori* stronger students (as reflected by analyzing their grades from the previous basic neuroscience course—see Section Descriptive statistics of students in Materials and Methods), thus their better grades in the advanced neuroscience course could not be attributed necessarily to the bonus-track process (as shown in the analysis of covariance presented in Section Grades of the Results). Since we did not have the final grades of the students that only participated in class, we cannot, at this point, claim that the contemplative methods contributed to improvement in class grades.

The 1-year follow-up quiz enabled us to investigate whether the brain investigations made a contribution to learning that went beyond providing students with a positive experience. A year after the completion of the course, students that participated in the bonus-track brain investigations were much more likely (even when final grade range in the course, percentage of classes attended, participation during class and concentration levels were used as covariates) to remember the course material relative to those that did not participate in the bonus-track. This finding suggests that extensive participation in the “personal brain investigations” not only contributed to the students' experience but also enriched and enhanced the representations of the class material leading to superior subsequent memory for the class information.

Our qualitative evaluation enabled a more fine-grained understanding of the process that the bonus-track students underwent. In this evaluation, we extracted themes that emerged in the written reflections of the bonus-track students. As students were not asked explicit questions in the final reports, the spontaneous emergence of a theme and its occurrence across individuals contains important insights regarding the contribution of the contemplative exercises to the students. First, we found that in accordance with the significant satisfaction scores that bonus-track students reported on the quantitative evaluations, the qualitative analysis revealed that most of them reported feeling positive emotions toward the contemplative investigations, such as interest (64%) and feelings of enjoyment and enrichment (55%). Only 11% reported negative emotions such as discomfort, boredom and unpleasant sensations (and the intensity of such emotions was small relative to the reports of positive feelings). Moreover, almost half of the bonus-track students (46%) reported feeling that the process contributed directly to their personal lives and provided them with new insights and discoveries (47%) regarding themselves. The feedback from one of the students exemplifies the experience of many bonus-track students: “I discovered a lot of new things about myself. I discovered a new perspective, instead of looking outwards, to focus inwards. That is something that I never experienced personally before. This is a very instructive and enriching experience. Most of the learning was about myself, about the rich world and the interaction between the two.”

In accordance with the quantitative results, the qualitative analysis of bonus-track students' summaries revealed that the contemplative exercises did not only add to students' general experiences but also contributed directly to their in-class learning. Close to one-third of the bonus-track students (27%) spontaneously reported that the investigations contributed to their understanding of the class material and to their learning experience. They mentioned that experiencing the theoretical material taught in class first-hand “provides depth to the course content” and “enables a completely different level of processing” beyond that provided by traditional reading or learning processes. Students in the bonus track mentioned that the personal brain investigations “connected the self to the theoretical course material.” Based on our personal teaching experiences, we can attest to the fact that psychology students (as well as students of other therapy-based professions) often find it hard to understand how biological courses relate to other courses in their undergraduate studies. Our findings suggest that contemplative exercises, such as the ones we proposed here, can be an effective way to bridge psychological and physiological processes and provide motivation to psychology students to learn about biological processes.

Notably, our qualitative analysis revealed that many bonus-track students reported being able to cultivate various contemplative skills during the semester. Bonus-track students reported an increased awareness of themselves and their environment (36%), the development of a non-judgmental attitude toward subjective experiences (30%) and the enhancement of their attentional skills (21%). Although the personal brain investigations did not explicitly cultivate these skills, this by-product was not unexpected. This is because the mindfulness skill set—namely “focusing attention, on purpose, in a particular way and non-judgmentally,” as Kabat-Zinn (1994) defined it—served as the basis for our contemplative approach. Thus, the fine-tuning of attention and awareness in the beginning of each investigation, and the non-personal, researcher-like stance bonus-track students were required to take upon themselves, enabled some of them to generalize these abilities to their daily lives.

Another skill, which approximately one-quarter of bonus-track students (26%) spontaneously reported cultivating during the process, was an improved ability to withstand the stresses and challenges of the semester. This finding is in-line with the large body of research showing that mindfulness practice can be employed for the sake of reducing stress, depression and anxiety symptoms (Hofmann et al., 2010). Some bonus-track students specifically addressed this benefit in their lab reports: one wrote that “it seems that when attention is focused to the body's posture, the emotional system remains stable and uniform, without oscillations in mood,” and another stated that “even just the polishing of the lens and the focusing on the breath helps relax the body and the thoughts.”

The fact that bonus-track students explicitly mentioned the cultivation of healthy mind skills is in accord with our quantitative findings that students claimed to want to use these contemplative tools in the future. The qualitative analysis also showed that bonus-track students not only identified these skills as being beneficial for their well-being and academic achievements during their stressful and challenging college year (see examples of reports in Section Developing a tool of Results), but also as being highly relevant for their future careers as therapists.

Taking the qualitative and quantitative evaluations together, we suggest that using contemplative exercises, such as the personal brain investigations we described here, can enrich neuroscience classes, help psychology students relate theoretical biological material to their personal experiences and professional lives, and contribute to learning and memory processes. In addition, these contemplative exercises can enable students to discover new insights about themselves, as well as cultivate contemplative tools and gain valuable skills to enhance their personal well-being and learning abilities.

Although most current advances in neuroscience pedagogy focus on incorporating technological tools to assist neuroscience teaching (Griffin, 2003; Estevez et al., 2010), several educators are seeking more experiential ways to help students connect the course content to their lives and increase motivation, engagement, and curiosity toward the theoretical class material (Stewart and Stavrianeas, 2008; Pollack and Korol, 2013). The “experimental contemplative lab” that we presented here provides students with hands-on access to mental and psychological processes related to class material and the possibility to explore and develop curiosity toward them. In some sense, contemplative methods make explicit the attempts made by students, often implicitly, to relate theoretical class material to their everyday life experiences. Importantly, such contributions of contemplative methods are not limited to undergraduate neuroscience courses for psychology students but can be implemented with a wide range of student ages and levels.

Several limitations of our evaluation methods should be considered. The first concerns the external validity of our results. Bonus-track students comprised 40% of the total student cohort and participation in the bonus track was based on the student's willingness to volunteer (and/or obtain the bonus points). In addition, bonus-track students had, on average, higher grades than the other students from the very beginning. Thus, it is not clear whether the positive effects that we found would also appear if all students were obliged to participate. However, it is important to note that approximately half of the students who participated in the bonus-track were below-average students (based on their grades the previous year, see Methods), suggesting the bonus-track is not only attractive to strong students.

Another limitation concerns the internal validity of our results. Differences between the bonus-track group and the other students could have been due to *a priori* dissimilarities between the groups. For example, we know that bonus-track students were, on average, better students. In addition, it is possible that the bonus-track students were also more focused, less stressed, more positive toward neuroscience material, and attended class more. These qualities may have enabled them to obtain more benefits from the contemplative process, as well as influenced learning and memory processes. In the analysis of the follow-up quiz we controlled for grade, class attendance, class participation and level of concentration, however we did not control for the other factors. Thus, it is possible that the difference we found on the quiz was a consequence of various qualities characterizing the bonus-track students and not the contemplative investigations themselves. Indeed, we do not have direct information regarding students' general attitude toward neuroscience classes which may have facilitated motivation to remember class material. However, regarding the issue of stress and focus, the fact that around one-quarter of bonus-track students spontaneously mentioned how the process had helped them withstand the stress and challenges of the semester, and increase attention and focusing abilities, suggests that many of the bonus-track students are not necessarily different in these aspects from students who did not complete the bonus-track.

Another limitation concerns the fact that the available data does not allow us to assess separately the effects of the in-class investigations and the effects of the bonus-track. Thus, we cannot conclude whether the in-class investigations, by themselves, can contribute to neuroscience teaching. In addition, the bonus track involved a bi-weekly personal feedback from the teacher, considerable written assignments and a bonus credit. All of these could have contributed more than the contemplative exercises themselves to the positive outcomes found for the bonus-track students. Although we cannot rule this out, the fact that several themes that arose from bonus-track students' spontaneous reports were explicitly related to the development of various contemplative skills (“increased awareness,” “non-judgment,” “attention and concentration”), suggests that the contemplative aspects of the exercises had a non-negligible contribution to the effects of bonus track.

A fourth limitation relates to the fact that the teacher (first author) was involved in the qualitative analysis, possibly introducing a bias in the analysis. Although this design is of course not optimal, we took caution to attenuate bias as much as possible (see Methods sections for details). We believe the existence of many thematic categories that describe difficulties and negative emotions toward the bonus-track program (such as “negative emotions,” “feeling of challenge,” feeling it was “static, exhausted itself,” “feeling a sense of duty”) suggest that the coders were not biased toward positive outcomes only. Finally, it is important to emphasize that the third coder who performed the final coding of all diaries was naïve to the goals of the manuscript.

An additional limitation of the study arises from the fact that the qualitative results may have been biased by the bonus-track students' desire to appease the teacher by submitting positive reflections in their final report. However, the large diversity of themes that extended beyond the examples given in the instruction of the assignment, and the fact that there were also several negative themes suggests students made an effort to reflect upon their experience and were not concerned about expressing negative thoughts and feelings. In addition, we believe that the non-judgmental stance of the teacher throughout the course encouraged students to be authentic regarding their reports of their experiences.

Despite these limitations, when viewing our results alongside the growing enthusiasm about the use of contemplative methods in higher education (Bush, 2011; Barbezat and Bush, 2013; Zajonc, 2013), the increasing interest in contemplative neuroscience tools (Varela et al., 1992; Lutz et al., 2007; Lutz, 2010; Desbordes and Negi, 2013) and the growing number of reports on the positive effects of mindfulness-based practices on students' achievements and well-being (Beauchemin et al., 2008; Shapiro et al., 2008; Mrazek et al., 2013), we believe the effects we are reporting are related to the contemplative exercises described here and can be generalized to a wide range of students.

Notwithstanding the important pedagogical contributions that the contemplative approach discussed here may have, it is important to note that its implementation may not be straightforward. The major reason for this is the fact that the success of this approach draws upon the teacher's personal internal resources, skills, and expertise. A teacher, who wishes to use contemplative tools in a neuroscience class, should be able to guide students during their personal brain investigations, propose questions for inquiry, and most importantly, help them acquire an investigative, non-judgmental stance. As can be seen from the qualitative analysis (Section Reference to technical aspects in Results), some students would have liked to have been provided with specific instructions regarding what to do and what to discover. However, the essence of the contemplative approach is that it enables students to investigate their experience as it evolves, incorporating all aspects of the experience into the inquiry process. It is therefore important that the feedback and guidance from the teacher is not goal-oriented and that it leaves space for unexpected discoveries. The ability to be in a state of equanimity and curiosity with everything that happens is a skill the teacher should cultivate; we recommend that the teacher spend time acquiring contemplative attitudes of inquiry and experiencing various personal brain investigations firsthand. Importantly, though the pedagogical approach described here is greatly inspired by mindfulness practice, it is not *a* mindfulness practice. In mindfulness practice one develops the mindfulness skill set (i.e., concentration and attention stability, perceptual clarity and equanimity) in order to nurture a deep awareness toward the relation between habitual reactions to mental and sensory events to psychological suffering, and the transient nature of all phenomenon (Grabovac et al., 2011). In the “experimental contemplative lab,” on the other hand, the mindfulness “skill set” is developed for a more modest aim—that of supporting the investigative process of specific mental processes relevant to class material. Although students may spontaneously generalize these abilities to gain wisdom and insights regarding their personal lives (for example, see Section Contribution and insights in Results), they are not explicitly led to do so. Thus, we believe that a teacher that has basic training in contemplative inquiry and has mastered to some level the mindfulness “skill set” can lead students through successful experiences of “personal brain investigations.” Although, gaining basic mindfulness skills is not challenging these days, given the wide availability of secular mindfulness-based courses (such as Jon Kabat-Zinn's 8-week MBSR course, Kabat-Zinn, 2013), this basic requirement naturally limits the contemplative approach to teachers who are willing and curious of these directions.

This point relates to the next one, which is the difficulty to provide “manuals” and ready-made kits for such a contemplative approach. Here, we provided only several examples of many possible personal brain investigations that can be conducted. We believe that the ability of a teacher to lead students through “personal brain investigation” relies on the teacher's personal experience and thus teachers should develop their own investigations. To do so, one should be reminded that relating theoretical class material to human experience is a natural process that occurs on a daily basis in psychology teaching courses. By bringing contemplative inquiry skills into this process, teachers can develop their own suggestions for contemplative investigations related to their specific class material and personal experiences. Additional suggestions for those who would like to implement a contemplative approach similar to ours in their class are provided in the Supplementary Section.

Finally, another reason why implementation of the contemplative approach may not be straightforward, relates to the fact it is a first-person method. As discussed above, first-person methods have significant shortcomings as a method of investigation due to the fact that they are highly subjective and difficult to verify (Overgaard et al., 2008). However, as more neuroscience studies attempting to use first-person tools are emerging (Lutz et al., 2002; Petitmengin et al., 2006; Dor-Ziderman et al., 2013), one can expect that contemplative neuroscience pedagogy will become more appreciated as well. We believe that the advantages and shortcomings of contemplative methods can be directly experienced in a pedagogical setup. In addition to discussing the limitations of contemplative methods as scientific tools in class, teachers should provide a means for students to directly experience the inter-subject variability in first-person reports (see student mentions of this issue in Section Group work in the Results). Opportunities to experience such inter-individual differences can be provided in a variety of ways, such as sharing observations in class, working in pairs or small groups, or uploading selected observations to a common virtual forum on the class website.

In summary, we have described here a contemplative approach for neuroscience teaching. Our pilot study suggests that this approach can contribute significantly to students learning and experience, as well as development of important learning skills such as attention and emotional regulation. Despite our initial results, further investigations are required to assess the effectiveness of such pedagogical approach. In addition, the implementation of such pedagogical approach is not straightforward as the prerequisites required from the teacher entail significant personal investment. Yet, such efforts are worthwhile. As the field of neuroscience moves toward the understanding of the most complex human experiences (e.g., Varela et al., 1992; Lutz, 2010; Desbordes and Negi, 2013) and is becoming more integrated in diverse fields of study, the ability to connect different levels of knowing is an important tool for future generations of investigators and therapists.

### Conflict of interest statement

The authors declare that the research was conducted in the absence of any commercial or financial relationships that could be construed as a potential conflict of interest.
