# Supplementary material for: Relation between contemplative exercises and an enriched psychology students' experience in a neuroscience course
Source: Front Psychol. 2014 Nov 18;5:1296. doi: 10.3389/fpsyg.2014.01296 (PMC4235268; doi:10.3389/fpsyg.2014.01296)
Supplement: Supplementary file 2 [file DataSheet1.DOCX]

Qualitative results

| Emotions | |
| --- | --- |
| *Enjoyment/Positive emotion* | "The exercises were very enjoyable for me, although not intuitive and easy" (Student 2). |
|  | "I enjoyed performing most of the tasks and noticing the changes and the things that are happening" (Student 84) |
|  | "I began the bonus track because of the bonus grade, but already from the first exercise I was exposed to a different world, to ten minutes that take me out of the crazy overloaded and restless life and just enables me to connect to myself, I enjoyed the almost daily work very much" (Student 69) |
|  | "the bonus-track was a daily pause for me, to stop and look at things that interest me but I don’t always have the time to investigate and learn about myself. The first time I did it was for the experience, the second investigation I did for the grade, but all the subsequent investigations I did because of enjoyment and curiosity." (Student 97) |
|  | "After a few exercises I became connected and I enjoyed the exercises." (Student 75) |
| *Interest* | "The brain investigation process was for me an interesting and intriguing experience. I was happy to discover every time interesting discoveries about myself and about brain processes" (Student 20) |
|  | "It was very interesting to discover new things about myself and about phenomenon that happen while directing attention in various ways and during daily activities that I preform (Student 39) |
|  | "The brain investigation was an interesting and exciting experience, during which many questions arose in me about how things really work." (Student 52) |
|  | "Since I never practiced this type of exercises before, it was very interesting and gave me a lot of insights." (Student 70) |
|  | "Along the way I came across some interesting and fascinating exercises that stimulated the desire to explore, understand and learn." (Student 79) |
|  | "The process was very interesting. I tried to explore and discover new things and there were times when I was indeed very surprised about what I found." (Student 86) |
|  | "It was very interesting, because for the first time I really looked into my feelings and as a result obtained some insights." (Student 88) |
|  | "The exercises were very interesting; they recruited my awareness to the daily behavior in terms of focus and attention." (Student 89) |
| *Challenge* | "Attention is a very elusive creature that depends upon situations in a very dynamic manner" (Student 12) |
|  | "I found it really hard for me to release control and focus my attention. Maybe this frustration is what led to my extra effort in the practice of the exercises" (Student 42) |
|  | "At first the tasks were very difficult; I found it hard to focus my attention." (Student 98) |
|  | "Exercises that involved attending to specific body organs were more difficult for me." (Student 100) |
| *Negative emotions* | "I felt discomfort" (Student 56) |
|  | "The total detachment from distractors and the silence created unpleasant and negative feelings" (Student 90) |
|  | "The experience was interesting in part, but I did not connect to many practices and they bored me (the practice was technical)." (Student 4) |
| *Contribution and insights* | |
| *Contribution to myself* | The practice helped me discover new things about myself and to get general insights" (Student 9) |
|  | "I discovered a lot of new things about myself. I revealed a new perspective, instead of looking outwards, to focus inwards. That is something that I never experienced personally before. This is a very instructive and enriching experience. Most of the learning was about myself, about the rich world and the interaction between the two". (Student 10) |
|  | "I felt that I was really trying to explore myself and the experience accompanied me from time to time in my daily experience". (Student 80) |
|  | "Although the aim of these investigations was not to look at myself and or to improve myself, I think they mostly contributed in this dimensions. I think that however I thought I was aware of my feelings beforehand, I am bit more aware of them today". (Student 11) |
|  | "It was fascinating; I discovered a lot of things about myself. For example, that I have a lot of patience and I can be focused, attentive and concentrated for a prolonged period of time". (Student 101 ) |
|  | "The most important discovery for me was related to the fact that it was the first time in my life that I got the opportunity to investigate myself." (Student 24) |
|  | "I am grateful for the opportunity to get to know myself more and experience it through the course." (Student 38) |
| *Contribution to my life as a student* | "The practice of brain investigations helped me understand the limitations of the attentional system" (Student 9) |
|  | "The exercises enabled a 'time out' for self-observation that was very needed in this over-loaded semester". (Student 31) |
|  | "I felt that the ability to breakout from the pressure of life in general, and school in particular, and to focus on these investigations, was a present and induced a more relaxed and calm atmosphere". (Student 24) |
|  | "Something to which I connected during the exercises is the fact that we as students of psychology are required to get very high grades and therefore manage very little to stop a moment and just breathe, feel the pulse, the feet, the position of the body, to put attention on our thoughts and feelings. The exercises allowed it." (Student 83) |
| *Contributions in general* | "It contributed to understanding the various physiological and psychological effects through actual experience" (Student 82) |
|  | "[There was] a legitimacy to look inside and sense things that I wouldn’t bring to mind if it wasn’t for the brain investigations. Just as we study the psyche, it is important to learn on our bodies and ourselves the connection between body and mind." (Student 62) |
| *Contribution to understanding of the material* | "I think that the brain investigations give a different perspective to the curriculum which is a unique pedagogic approach" (Student 82) |
|  | "In general I believe that a good learning process needs to include some personal experience that relates to the learned material, and I think that this process enables this. Such an experience provides depth to the contents of the course and enables a completely different learning experience from the common one. We are used to receiving information from the teachers and books; such a personal experience enables a completely different level of processing. I hope there will be more experiences such as these in other courses too!" (Student 14) |
|  | "This was really an enriching and enjoyable experience, which connected the self to the theoretical course material." Student 65 wrote: "I really enjoyed some of the exercises, and they also contributed to the understanding of the materials in other courses" (Student 16) |
|  | "The connection between the brain exercises and the material taught in class that week is very important and it worth preserving it. It helps connect the theoretical knowledge and the practice and highlights the physiological contexts in daily life; this is a unique and interesting perspective" (Student 71) |
| *Contribution to the learning experience* | "This training contributed to me personally and made ​​me think about a lot of things that I never thought about in the past. It really added to the learning experience in this course and helped in the understanding of the material" (Student 22) |
|  | "It contributed to learning. To experience something is not just like reading or learning about it" (Student 41) |
|  | "I believe the experience adds an additional and different dimension to the course, and enables students who are interested to explore what they learned in the course for themselves" (Student 16) |
|  | "It is nice to have the opportunity to investigate things beyond the "dry" material taught in class" (Student 37) |
|  | "All in all, I enjoyed the experience, we all appreciate having the opportunity to experience what we learn, this is not something that happens in other places" (Student 36) |
|  | "I think the idea to enable an experiential dimension along the course is positive and very much needed especially in a psychology degree where everything is so theoretical. This enables reflecting on the class material beyond reading class summaries and enables feeling connected with the class material. They should consider doing this in other courses too" (Student 28) |
| *Discovery of new dimensions and insights* | "I discovered in myself several aspects that I never met before, even when some were hard for me" (Student 54) |
|  | "Now, every time that I engage in sports activities I turn my attention to the pulse in different body organs, at rest and during activity". (Student 64) |
|  | "It was the first time I noted the actions that I do, the physical activity that is usually automatic, I felt every movement and investigated how they are done. I think I had experiences that I wouldn’t have had an opportunity to investigate in any other place". (Student 17) |
|  | "I think this exercise contributed also personally and caused me to think about things I never thought about in the past." (Student 22) |
|  | "The personal investigation track helped ​​me understand several things that can't be understood based on studying the material only, this track had a complementary value beyond that of classes and exercises". (Student 93) |
| *Dynamics* | |
| *Development*  *Static, exhausted itself* | "I think that the exercises had a developmental trajectory, therefore there is not a single exercise that I would give up" (Student 8) |
|  | "In the beginning it was strange to be in the "shoes of investigators" but as the exercises advanced my feeling changed and I felt that with each investigation I was discovering new things about myself I wasn’t aware of beforehand" (Student 13) |
|  | "In the beginning I felt discomfort with the exercises, didn’t think I would connect to them even later and did the things from a feeling of obligation. However, slowly, I succeeded in connecting to them more and even had interesting findings that caused me to notice my attention and feelings even during the day and not only during the exercises" (Student 34) |
|  | "With time, the reports about pain decreased and eventually vanished. I did not realize it until now that I summarize my research. I have developed a certain skill". Student 67 noted: "While summarizing the tasks that I submitted, I was amazed to realize the process that I went through week after week" (Student 21) |
|  | "At first the tasks were very difficult, I found it hard to focus my attention, but with time and practice, the tasks became easier and enjoyable". (Student 98) |
|  | "At first I didn't realize how such simple exercises can bring great insights. However, with the passage of time and while performing more and more exercises, I was surprised to find how much you can learn from something so simple." (Student 58) |
|  | "The first week I arrived with great cynicism about the subject, but with time I connected very much to the exercises and I felt I learnt a lot about myself." (Student 77) |
|  | "Most exercises were really interesting, but there were times that I felt a sense of exhaustion and did not understand why we have to practice the same exercise again." (Student 3) |
|  | "The content of the trainings was very similar most of the times, so the exercises became sometimes tedious and ineffective."(Student 9) |
|  | "Sometimes there were tasks from week to week which were very similar to each other and it started to be a little boring." (Student 36) |
| *Sense of duty* | |
| *Feeling of a duty* | "There were times when I was stressed and performed the exercises out of duty, but not in most cases". (Student 72) |
|  | "The experience was mostly interesting, but at its end I felt I was doing it from a feeling of duty." (Student 53) |
|  | "There were several times when I felt a sense of duty and therefore, in those times I connected only during exercise itself." (Student 93) |
| *Duty led to positive results* | "At the beginning of the process the exercises were performed as an obligation, as part of the bonus track, doing the minimum required. However, with time I came across fascinating and interesting exercises that stimulate the desire to explore, understand and learn" (Student 79) |
|  | "Without the [repeated] practice I would have never performed exercises of this type, because normally we relate to the body as a machine without needs". (Student 68) |
|  | "It was hard for me to sit 4 nights and look at myself. This is something that I personally find challenging, since I try to do other things most of the time. Thus, after a few weeks I felt I was doing it out of a sense of duty. But when we got to the investigations that focused on states of consciousness, the interest came back to me and the sense of duty disappeared". (Student 11) |
|  | "I enjoyed the bonus track, even only because of the commitment that made me allocate a few gracious minutes a day that were only mine". (Student 73) |
|  | "Sometimes there is no other choice, for a process to be instructive and consistent you need something external to make you do it, and if it wasn’t obligatory I guess I would have given in to myself occasionally." Student 46 commented: "I understand that it is important to practice a large number of times in order to obtain results and insights". (Student 32) |
|  | "I would not give up to the fact that weekly assignment had to be submitted on the same day and at the same time every week. The fact that the investigations were performed continuously for more than two months, made the process deep and interesting." (Student 83) |
| *Developing a tool* | |
| *Increased awareness* | "It is nice to raise the level of awareness to things that without the exercises I wouldn't necessarily be aware of" (Student 5) |
|  | "My ability to focus attention increased or alternatively my awareness to it was raised". (Student 18) |
|  | "it was interesting to see that when one investigates something abstract and disintegrates it to its parts, it becomes simpler to understand." (Student 12) |
|  | "I think this is a process of looking inside that is beyond the investigations, but also for my daily experiences, understanding what influences me more and what less, getting to know myself and how my attention works." (Student 33) |
|  | "When I started practicing every week, I knew that I was entering ten minutes of silence before beginning the exercise. It was very interesting to see the change in me." (Student 66) |
|  | "I realize I have developed a skill…now, occasionally, there are moments that I suddenly notice details I have not noticed in the past. This happens every few days, but it is interesting and also gives a good feeling." (Student 21) |
|  | "I really noticed that some exercises brought me positive feelings while others negative emotions." (Student 99) |
|  | "The exercises provided me with a different perspective, helped me become aware of myself, of my body, of my breath, of my movement, of how various stimuli distract me from experiencing my inner experiences. How much I don’t know my body and how much I miss when I don’t listen to it. The body knows my situation even before I understand it. When stressed the body signals the overload burden and the feeling of heaviness, and when relaxed the body sends pleasant feelings". (Student 10) |
| *Non-judgment* | "I let myself investigate all the small things that constitute me, without trying to fix it. To try as much as I can to investigate, to focus and polish the lens." (Student 45) |
|  | "I became less and less judgmental towards myself, even in advanced exercises which I did not succeed. This is an achievement by itself; to be in the process and not necessarily reach the target. The result is not less interesting." (Student 94) |
|  | "I learned how to observe myself in a more objective way, although it is not simple and one needs to look out for the subjective interpretations that constantly enter." (Student 35) |
|  | "I think that everyone, but especially psychology students, who want to understand the human mind and its internal processes, should go through this route, and thus obtain a different perspective about ourselves and insights about our daily experience." (Student 95) |
| *Attention and concentration skills* | "Although I am not diagnosed with ADHD, many times I feel that it very difficult for me to concentrate and that maybe I suffer from attentional deficits. The exercises gave me a different perspective, helped me become more aware of myself, my body, my breathing, my movement and to understand how all the distracting stimuli mask my ability to attend my inner experiences" (Student 10) |
|  | "I feel that I have acquired self-questioning tools that allow me to influence and control my attention" (Student 41) |
|  | "With time, attention allocation become more automatic, without relation to the specific instructions for the week." (Student 19) |
|  | "My ability to focus attention improved or at least rose to a higher level of awareness." (Student 18) |
|  | "The order of the exercises was meaningful, since as the weeks past I felt I became more skillful in focusing attention, and I could do more complex tasks that I assume I couldn't have done in the first weeks." (Student 39) |
|  | "The most important thing that I would keep is the sharpening of attention before each exercise. I think that it is important to focus first on the breathing, and by doing so, enter the process incrementally to reach a better focusing of attention and be able to discover much more interesting things." (Student 85) |
|  | "I feel I have developed some kind of meditative skill, which is still in its infancy, but it gave me a taste of this world, and left in me a longing to come back and investigate it further." (Student 23) |
|  | "The brain investigations process caused me to understand how important it is for me to focus attention on the things that I am occupied with in order to succeed in it and not do several things at the same time". (Student 25) |
| *Relaxation and stress reduction skills* | "I am an anxious person by nature. Me and my close relatives can attest that lately the exercises helped me significantly in relaxing." (Student 82) |
|  | "Unfortunately, due to the large load and amount of tasks that we get, we do not always have the time to stop and breathe and be with ourselves. The exercise was a good attempt to allow us such quality time". (Student 47) |
|  | "I felt this helped me to deal with stress and various feelings. In addition, it was interesting to connect to myself and just be in peace and serenity with myself for 10 minutes every day." (Student 29) |
|  | "This enabled me to realize a few things about myself, such as the importance of training attention and the importance of rest and relaxation from the daily tasks." (Student 11) |
| *Reference to technical aspects* | |
| *Guidelines* | The practice at the beginning of class, was very powerful and gave us an introduction to what is expected from us, how to work during the week" (Student 33). |
|  | "I would leave [for future bonus-track exercises] the slight vagueness in which the guidelines were given…and the explanations about polishing and focusing the lens in the first weeks that helped perform the exercise subsequently" (Student 61). |
|  | "Despite being confused many times, I understand now that the opaqueness of the exercises contributed" (Student 40). |
|  | "I think that the process could give even more if there were more precise guidelines. What should we do? What should we search for? At first I felt a little lost and maybe the process would be more effective if there was a tighter guidance." (Student 43) |
|  | "I would love to understand more in depth the theoretical and scientific basis underlying the tasks that we performed" (Student 64). |
|  | "Sometimes the guidelines were too general, and there was a need for more explanations to understand what to do…In this case I did what I understood, but wasn’t sure it was the right thing" (Student 1) |
|  | "It is important to emphasize from the start that we are not required to investigate ourselves in a personal manner… only after you gave me feedback I understood I need to focus on general processes and not personal problems." (Student 17) |
|  | "I think I'd sharpen more the instructions, especially at the first few weeks and I'd love to get a bit more detailed feedback." (Student 77) |
| *Feedback* | "I liked the fact that you gave feedback, even just the word "thank you"; it helped me to know that I am doing things right and that you really care and are interested in my investigations" (Student 69). |
|  | "The most substantial thing was the feedback. An integral part of my will to report was to hear your opinion, is this what you meant? Is this something new? How can I improve? etc." (Student 53). |
|  | "I would continue [for future students] the persistence to investigate the processes with an "investigator's eye" and not from a psychological perspective." (Student 74) |
|  | "I would have been happy if you could provide a deeper feedback of the observations that I wrote and shared". (Student 74) |
|  | "I think I would prefer receiving feedback every week." (Student 53) |
|  | "I would add feedback in the form of questions. Once you asked me what I meant when I said 'soul'. This question made ​​me think beyond, and contributed to my process."(Student 95) |
| *Higher weekly frequency* | "The fact this is an exercise that is required to do several times a week is important as it provides an opportunity to enter 'into it'" (Student 14) |
|  | "Sometimes I felt two repeats would be enough but other times I felt I could have investigated and discovered more" (Student 19). |
|  | "I wouldn’t change the fact you need to report 4 observations a week because every observation enables you to look at the exercise from a different perspective and in that way discover more" (Student 59) |
|  | "I would stay with four weekly observations since there are real differences when you perform the exercise in different places, different moods, different hours, etc." (Student 78) |
| *Lower weekly frequency* | "I think that it was enough to report only three times a week since the fourth time was already too much of a burden and sometimes made ​​me forget the main reason for doing the exercise" (Student 18) |
|  | "Maybe we should start from four exercises per week to understand the issue of inner concentration and attentional practice, and then from the 6th week onward to reduce the number to three" (Student 43) |
|  | "Although the exercises are only 10 minutes a day, I felt the requirement to do them four times a week was a bit exaggerated" (Student 48) |
|  | "I would suggest more flexibility in the number of times per week to perform the exercises. Instead of having to exercise four times a week, I would allow a range between three and six times, in such a way there will be less a feeling of a rigid framework and more opportunity for free will" (Student 55) |
| *Group work* | "I would set at least two exploration tasks to work on pairs so we can see how the same questions and guidelines are interpreted and experienced differently by each person. It is important that we also see the inter-individual differences" (Student 97) |
|  | "I would have been happy if once all the participants in the bonus-track can meet and share experiences, I think this could have been interesting"(Student 14) |
| *Informal practice in everyday life* | "I think the informal practice helped me much as it enabled me to see how the investigations are reflected in daily life." (Student 21) |
|  | "It is important [for future students] to continue with the informal practices that for me, at least, enabled the most interesting discoveries." (Student 50) |
